# 

**Published:** 1877-06

**Authors:** 


### CONTENTS:

ORIGINAL COMMUNICATIONS:

A Simple Means of Securing the Best Posture for the Leg in Case of Compound Fracture. By Dr. H. Banga, Chicago ..... 485

Hysterical Aphonia. By G. Whitefield Ward, A.M., M.D. ..... 496

An Improved Method of Performing the Radical Operation in Empyema. By E. Fletcher Ingals, M. D. ..... 505

Scarlet Fever and the Board of Health. By Henry M. Lyman, M. D. ..... 514

The Limits of the Opitcal Capacity of the Microscope. By Lester Curtis, M. D., Chicago ..... 526

CLINICAL REPORTS:

Rush Medical College Clinic ..... 531

Extract from Notes of Obstetric Practice in the Chicago Hospital for Women and Children since its Opening, May 8th, 1865, to May 8th, 1877 ..... 534

REPORTS OF SOCIETIES:

The Convention of the Illinois State Medical Society ..... 536

REVIEWS AND BOOK NOTICES ..... 550

MEDICAL NEWS AND ITEMS ..... 555

EDITORIAL NOTICES ..... 563

BOOKS AND PAMPHLETS RECEIVED ..... 566

CORRESPONDENCE ..... 566

SUMMARY OF PROGRESS IN MEDICAL SCIENCES:

I. Surgery ..... 567

II. Therapeutics ..... 573

III. Ophthalmology ..... 576

ANNOUNCEMENTS FOR THE MONTH ..... 580

